# Biodiversity data mining from Argus-eyed citizens: the first illegal introduction record of *Lepomis macrochirus macrochirus* Rafinesque, 1819 in Japan based on Twitter information

**DOI:** 10.3897/zookeys.569.7577

**Published:** 2016-02-26

**Authors:** Yusuke Miyazaki, Akinori Teramura, Hiroshi Senou

**Affiliations:** 1 Kanagawa Prefectural Museum of Natural History, 499 Iryuda, Odawara-shi, Kanagawa 250-0031, Japan; 2 Faculty of Marine Science, Tokyo University of Marine Science and Technology, 4-5-7 Konan, Minato-ku, Tokyo 108-8477, Japan

**Keywords:** Biotope, Centrarchidae, environmental education, Invasive Alien Species Act, recreational fishing, tweet, voucher

## Abstract

An apparent illegal introduction of *Lepomis macrochirus macrochirus* from Yokohama City, Kanagawa Prefecture, Japan, is reported based on a juvenile specimen and a photograph of two adults collected on 14 June 2015 and deposited in the Kangawa Prefectural Museum of Natural History. The specimens and photographs were initially reported on the internet-based social networking site, Twitter. Two specimens of *Carassius auratus*, including an aquarium form, were also reported at the same locality and date, suggesting that the illegal introductions originated from an aquarium release. Our report demonstrates an example of web data mining in the discipline of Citizen Science.

## Introduction

Rapid biodiversity decline is a serious problem requiring a global response. The spread and resultant establishment of invasive non-native species is one of the most critical contributing factors to biodiversity decline (Puth and Post 2005; Blackburn et al. 2011), and the detection of invasive species is required globally to safeguard biodiversity.

Japan’s “Invasive Alien Species Act (IASA)” was established in 2005 under the Basic Biodiversity Act, within the Environmental Act (Oikawa 2010). The IASA prohibits the introduction and spread of Invasive Alien Species (IAS) as defined by the law in Japan. That is, the breeding, cultivation, storage (in either natural or artificial conditions), transportation, transfer, delivery, importing and releasing from an already established place to anywhere else including artificial habitats, planting and dispersing to outdoors of IAS are strictly prohibited. Any person who violates the IASA faces a fine of <3 million yen or <3 year of penal servitude, while a corporation that transgress faces a fine of <100 million yen (Study Group on Impacts and Managements of Alien Species 2008).

The first suggested illegal introduction under the IASA was a report of the centrachid fish *Micropterus salmoides* (Lacepède, 1802), which remains, unfortunately, one of the most popular recreational fishing targets in Japan. The species was apparently illegally released into an irrigation pond in Ichinoseki City, Iwate Prefecture, during October 2007–May 2008 (Miyazaki 2010). The second report also recorded the introduction of the same species in the same prefecture. This IAS was illegally released into three irrigation ponds of Oshu City, Iwate Prefecture, between 2008 and 2009 (Tsunoda et al. 2011). Following this, on 19 August 2009, the first arrestee under the IASA, a black bass fishing fan who had transferred two live specimens of *Micropterus salmoides*, was reported by several mass media outlets such as Jiji Press, The Asahi Shimbun, and Nara Newspaper (e.g., Miyazaki 2010).

Another IAS, *Lepomis macrochirus macrochirus* Rafinesque, 1819, also belongs the family Centrarchidae. The first recorded introduction of the species in the natural waters of Japan was in 1963 (Matsuzawa and Senou 2008; Senou and Hayashi 2013). The origin of all Japanese populations of the species is the 18 individuals donated from the Shedd Aquarium (Mississippi River population) by the Chicago mayor of October 1960 (based on the analyses of mtDNA of the Japanese populations: Kawamura et al. 2004, 2010); these were bred at the Freshwater Fisheries Research Laboratory of Fisheries Agency, Japan. After the donation, they were introduced to four man-made lakes in Miyazaki, Kochi, and Tokushima Prefectures during 1963–1964 ahead of the ceremonial introductions by the Japanese prince in April 1966 to Lake Ippeki, Shizuoka Prefecture. The species was then introduced widely across Japan for the purposes of commercial fisheries and ceremonies (Nakai 2002). Since the 1970s, this species has been often released together with *Micropterus salmoides* because several columns in Japanese recreational fishing magazines encouraged the widespread and combined introductions of the two species (Nakai 2002; Matsuzawa and Senou 2008). In addition, *Lepomis macrochirus macrochirus* was unintentionally spread during the widespread release of *Plecoglossus altivelis altivelis* (Temminck & Schlegel, 1846) from Lake Biwa; the latter was commercially stocked in Lake Biwa and then widely released during the 1970s–2000s (see also Miyazaki et al. 2015a). Both *Micropterus salmoides* and *Lepomis macrochirus macrochirus* had spread to all Japanese prefectures by 2001 (Kiriu 1992; Hayashi 2002; Maruyama 2002; Nakai 2002). However, the former two examples of illegal introductions were of *Micropterus salmoides* and, despite the circumstantial evidence of the illegal introduction of *Lepomis macrochirus macrochirus*, this illegal activity has not been previously reported from Japanese waters.

Here, we detail the first apparent introduction of *Lepomis macrochirus macrochirus* in Japan since the IASA was adopted.

## Integrating information from a student’s tweet to museum collections

**Figure 1.**
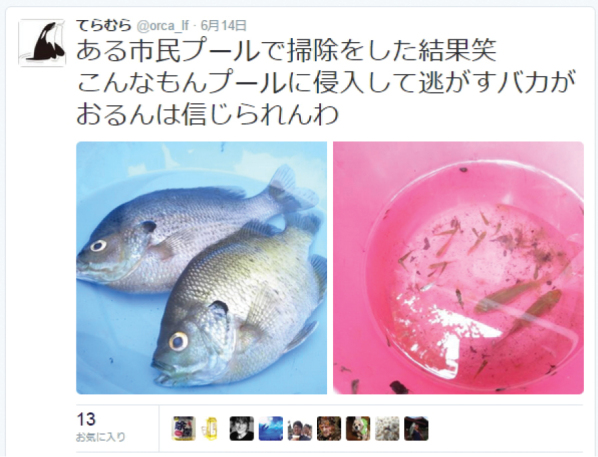
A tweet including two fish images posted by AT on 14 June 2015. The image on the left shows two adults of *Lepomis macrochirus macrochirus*, while the one on the right shows juveniles of *Lepomis macrochirus macrochirus* and two young of *Carassius auratus* (however, the specimens in the latter image are not able to be identified even to family level from the image alone). The comments are translated into English as follows: “The results of a pool cleaning. I cannot believe the foolishness of the person who introduced these fish into the pool.” https://twitter.com/orca_lf/status/610088107476516864 [Accessed on 25 November 2015]

On 14 June 2015, the second author (AT), an undergraduate student of the Tokyo University of Marine Science and Technology, tweeted a comment along with two images on a social networking service, Twitter, via the internet (Fig. 1). It stated that he had identified *Lepomis macrochirus macrochirus* from a public outdoor swimming pool and was obviously surprised that people could be so irresponsible as to release an invasive species. The first author (YM) saw the tweet, and contacted AT with the idea of publishing the information as a scientific report.

**Figure 2.**
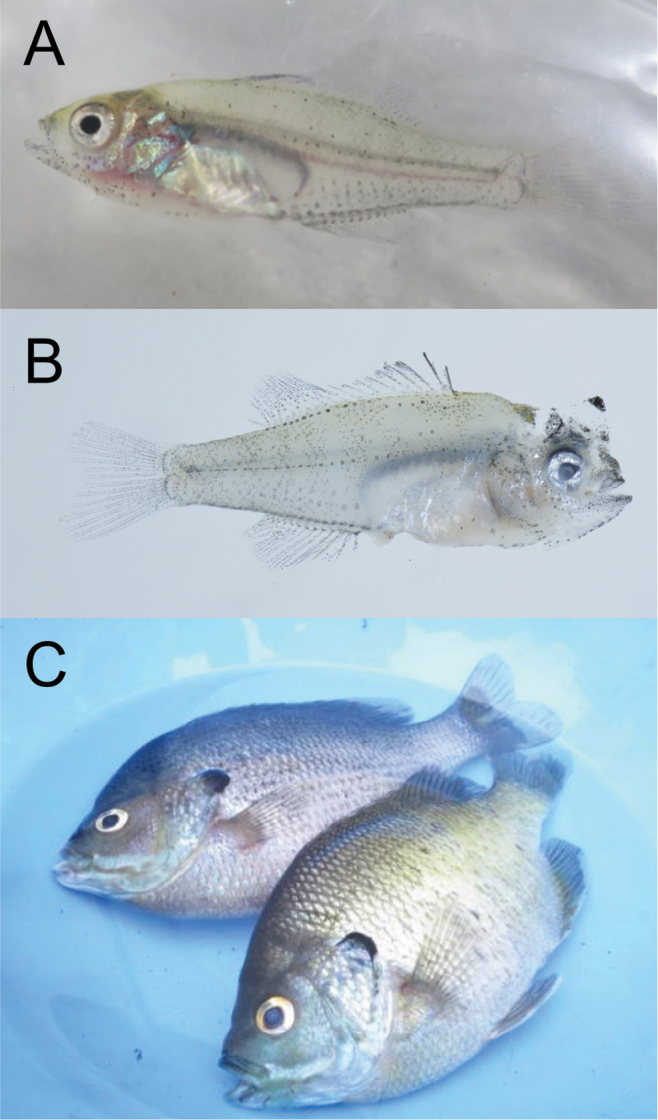
Photographs of *Lepomis macrochirus macrochirus* collected from the outdoor pool of Chigasaki Park, Yokohama City, Kanagawa Prefecture, Japan on 14 June 2015. **A** voucher specimen, juvenile, KPM-NI 38654 (photo: KPM-NR 164120 by AT), 22.6 mm SL **B** voucher specimen, juvenile (right side), KPM-NR 108928 by YM (the same individual as KPM-NI 38654) **C** adult specimens, photograph, KPM-NR 164118 by AT.

**Figure 3.**
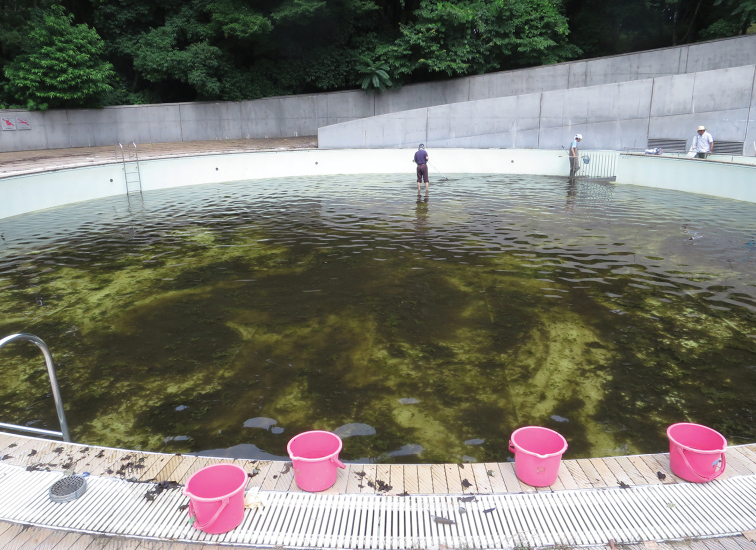
Photograph of the outdoor pool of Chigasaki Park, Yokohama City, Kanagawa Prefecture, Japan.

As a result, we deposited a juvenile specimen of *Lepomis macrochirus macrochirus* collected from the artificial outdoor pool in Chigasaki Park, Yokohama City (35°32'20.8"N, 139°34'54.0"E; 40–50 m at middle latitudes), on 14 June 2015 in the Kanagawa Prefectural Museum of Natural History, Japan (KPM-NI 39654; Fig. 2A, B). The pool has been isolated from natural waters since its construction (Fig. 3). Additionally, the public museum catalogs and stores fish images as well as specimens (Miyazaki et al. 2014), so we also registered the above specimen’s photographs (KPM-NR 108928, 164120; Fig. 2A, B), and the original image of two *Lepomis macrochirus macrochirus* adults that was posted on the internet via Twitter on the image database (KPM-NR 164118; Figs 1, 2C). Based on the above voucher specimen and photographs, we identified the specimens as the invasive fish species, *Lepomis macrochirus macrochirus* (Scott and Crossman 1973; Ross et al. 2001; Yamamoto and Yodo 2014), noting the following external morphological characters. In adults (photograph): mouth small; posterior end of maxilla not reaching anterior margin of eye; posterior part of opercle with flap; deep body; dorsal, pelvic and anal fins with spines. In juvenile (specimen): D X, 10; A III, 10; P_1_ 13; P_2_ I, 5; dark transverse bands on body.

**Figure 4.**
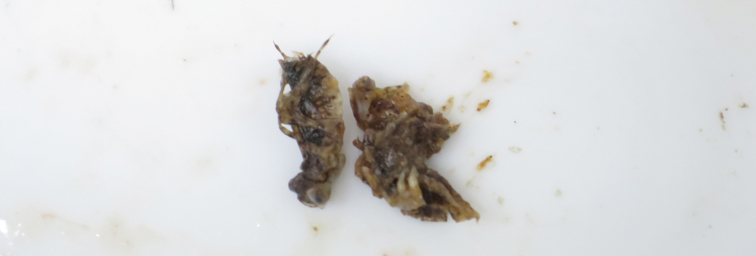
A portion of gut components of *Lepomis macrochirus macrochirus* collected from the outdoor pool of Chigasaki Park, Yokohama City, Kanagawa Prefecture, Japan, on 14 June 2015. Dragonfly nymphs, *Sympetrum* sp., were included.

We also identified some of the gut contents of an adult *Lepomis macrochirus macrochirus* as dragonfly nymphs of the genus *Sympetrum* (Fig. 4). This identification follows Ishida (1996) and is based on the following characters: laterally long and inverted trapezoid head, large and prominent compound eyes, and presence of lateral spines in abdominal segments 8–9.

**Figure 5.**
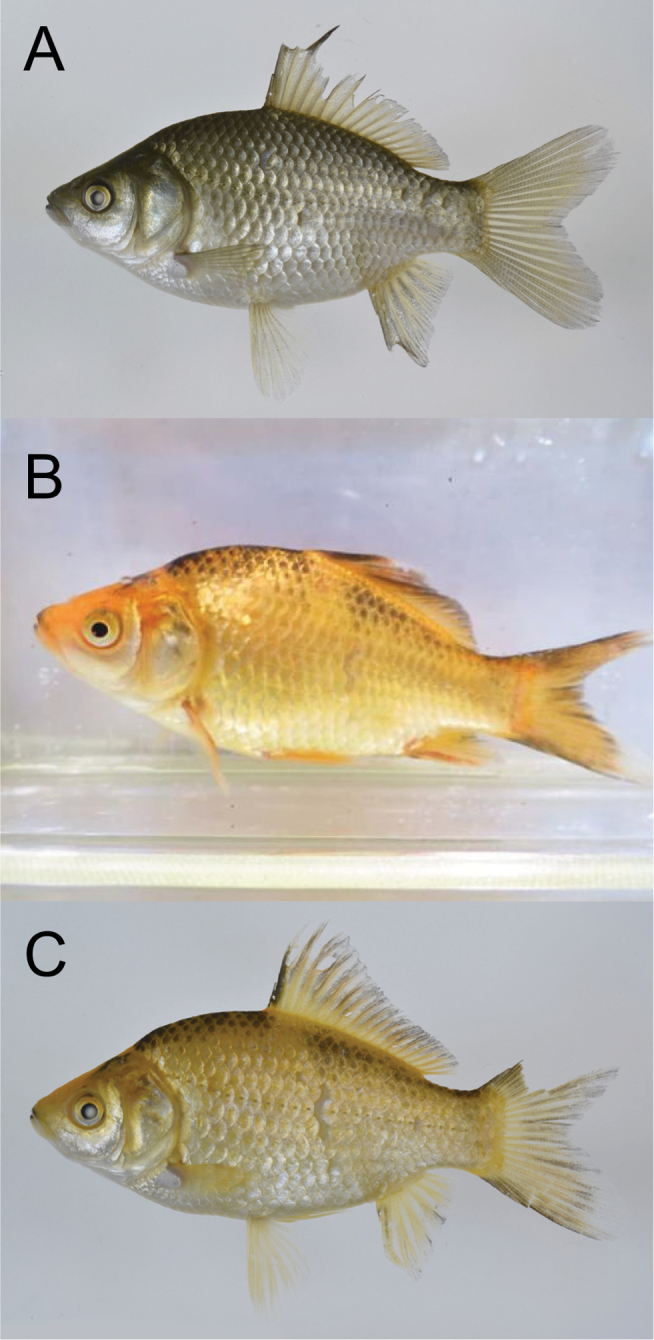
The voucher specimens and photographs of *Carassius auratus* collected from the outdoor pool of Chigasaki Park, Yokohama City, Kanagawa Prefecture, Japan, on 14 June 2015. **A** voucher specimen, KPM-NI 38655 (photo: KPM-NR 108929 by YM), 64.9 mm SL **B** voucher specimen (live), KPM-NI 38656 (photo: KPM-NR 164119 by AT), 61.3 mm SL **C** voucher specimen (fresh), KPM-NR 108930 by YM (the same individual as KPM-NI 38656).

In addition, we also deposited two specimens and photographs of the goldfish, *Carassius auratus* (Linnaeus, 1758), which were collected and recorded along with the *Lepomis macrochirus macrochirus* specimens (Fig. 5). The goldfish specimens had the following characters (compared with Japanese *Carassius* spp.): higher body depth (45.1–46.8% of standard length), fewer gill rakers (37–40), fewer numbers of pores in lateral line (each 28), longer fins, and silvery–golden and/or yellowish body colors.

We note, in passing, that *Carassius auratus* is native to China and the Korean Peninsula but not the Japanese archipelago (Kalous et al. 2012). Specimens from Japan have been comfirmed by mtDNA analyses to have originated from Chinese populations (Komiyama et al. 2009). That is, it is an exotic non-native species in Japan. Although molecular analyses (Takada et al. 2010; Sakai et al. 2011; Kalous et al. 2012) strongly suggest that some Japanese *Carassius* spp. are endemic, the species’ morphological identifications have been confused, except for *Carassius cuvieri* Temminck & Schlegel, 1846 (see also Taniguchi 1982; Hosoya 2002, 2013; Iguchi et al. 2003; Suzuki et al. 2005; Yamamoto et al. 2010; Saitoh and Uchiyama 2015). Generally, Japanese taxonomists have labelled the Japanese endemic taxa as *Carassius langsdorfii* Temminck & Schlegel, 1846 or *Carassius buergeri* Temminck & Schlegel, 1846 and their subspecies, but their classification schemes have not been published in the English literature.

## Discussion

In 2014, the public outdoor pool where the IAS and goldfish specimens were found operated between 12 July and 7 September; this suggests that *Lepomis macrochirus macrochirus* and *Carassius auratus* were introduced sometime between 8 September 2014 and 14 June 2015. This report is the first circumstantial evidence of the illegal introduction of *Lepomis macrochirus macrochirus* in Japan based on the IASA after it was enacted on 1 June 2005.

Generally, outdoor swimming pools that are not in operation during autumn and spring usually function as spawning and nursery habitats for native aquatic insects such as dragonflies and diving beetles (e.g., Lee et al. 1998; Taguchi et al. 2000; Ido and Goto 2002; Takeyama et al. 2002). Therefore, they are sometimes used for environmental education activities, utilizing them as a biotope. The present report is a case in point, where the pool was being cleaned and its organisms observed. The invasion of *Lepomis macrochirus macrochirus* frequently causes population decline of aquatic insects (sometimes including threatened species). In fact, AT found dragonfly larvae or juveniles in the gut components of individuals of the invasive fish species collected from the pool (see Fig. 4).

The discovery of *Carassius auratus* at the same locality and date as the IAS, suggests that the fishes might have originated from an aquarium fish release from a local shop or an aquarist who no longer wanted them. However, based on the IASA, keeping *Lepomis macrochirus macrochirus* in a home aquarium is illegal, and of course its release is also strictly prohibited. Any specimens collected from Japanese waters should be destroyed. From a bioethical viewpoint, several groups (particularly bass-fishing fans) do not support such killings (e.g., Mizuguchi 2005). However, they are required only because of the irresponsible actions of people who release invasive species from controlled environments such as aquaria to nature. Without such releases, the killings would be unnecessary. Community awareness of this issue needs to be improved, and widespread reporting of cases such as this one will help.

More than 10 years have passed since the IASA was enacted, and the illegal introductions of *Micropterus salmoides* and *Lepomis macrochirus macrochirus* have possibly occurred mostly via the younger generation who are not aware of the law. It has been pointed out that they probably contribute to the wider distribution of invasive species such as those discussed here (e.g., Yodo and Iguchi 2004; Miyazaki 2010). Ideally, the IASA should be advertised again to all Japanese generations.

Web data mining has been rapidly developing over recent years, and its potential continues to expand (Liu 2011). This report shows an example of web data mining in the discipline of Citizen Science, as similarly shown by Miyazaki et al. (2015b). It demonstrates the “accidental” crowdsourcing approach but not the “systematic” crowdsourcing approach such as iSpot (Silvertown et al. 2015).

## References

[B1] Blackburn TM Pyšek P Bacher S Carlton JT Duncan RP Jarošík V Wilson JRU Richardson DM (2011) A proposed unified framework for biological invasions. Trends in Ecology and Evolution 26: 333–339. doi: 10.1016/j.tree.2011.03.023 21601306 10.1016/j.tree.2011.03.023

[B2] Hayashi M (2002) Centrarchidae. In: Nakabo T (Ed.) Fishes of Japan with Pictorial Keys to the Species, English Edition Tokai University Press, Tokyo, 745.

[B3] Hosoya K (2002) Cyprinidae. In: Nakabo T (Ed.) Fishes of Japan with Pictorial Keys to the Species, English Edition Tokai University Press, Tokyo, 253–271 + 1464–1467.

[B4] Hosoya K (2013) Cyprinidae. In: Nakabo T (Ed.) Fishes of Japan with Pictorial Keys to the Species, Third Edition Tokai University Press, Hadano, 308–327 + 1813–1819 [In Japanese]

[B5] Ido T Goto H (2002) A case study on establishment and use about school bio-top—case study on the Eco-up enterprise by dragonfly pool at Yokohama City. Journal of Architecture, Planning and Environmental Engineering (Transactions of AIJ) (554): 213–218. [In Japanese with English abstract]

[B6] Iguchi K Yamamoto G Matsubara N Nishida M (2003) Morphological and genetic analysis of fish of a *Carassius* complex (Cyprinidae) in Lake Kasumigaura with reference to the taxonomic status of two all-female triploid morphs. Biological Journal of the Linnean Society 79: 351–357. doi: 10.1046/j.1095-8312.2003.00196.x

[B7] Ishida K (1996) Monograph of Odonata Larvae in Japan. Hokkaido University Press, Sapporo, 447 pp. [In Japanese]

[B8] Kalous L Bohlen J Rylková K Petrtýl M (2012) Hidden diversity within the Prussian carp and designation of a neotype for *Carassius gibelio* (Teleostei: Cyprinidae). Ichthyological Exploration of Freshwaters 23: 11–18.

[B9] Kawamura K Yonekura R Ishikawa M Katano O (2004) Genetic characteristics of a bluegill sunfish, *Lepomis macrochirus*, in Japan and Korea in the restriction fragment length polymorphism (RFLP) of mitochondrial DNA. Fish Genetics and Breeding Science 33: 93–100. [In Japanese with English abstract]

[B10] Kawamura K Yonekura R Ozaki Y Katano O Taniguchi Y Saitoh K (2010) The role of propagule pressure in the invasion success of bluegill sunfish, *Lepomis macrochirus*, in Japan. Molecular Ecology 19: 5371–5388. doi: 10.1111/j.1365-294X.2010.04886.x 21044195 10.1111/j.1365-294X.2010.04886.x

[B11] Kiriu T (1992) Distributions and habitat of the bluegill. In: National Federation of Inland Water Fisheries Cooperatives (Ed.) All about Black Bass and Bluegill: A Report of Project Contracted Research on the Control of Exotic Fishes. National Federation of Inland Water Fisheries Cooperatives, Tokyo, 89–103. [In Japanese]

[B12] Komiyama T Kobayashi H Tateno Y Inoko H Gojobori T Ikeo K (2009) An evolutionary origin and selection process of goldfish. Gene 430: 5–11. doi: 10.1016/j.gene.2008.10.019 19027055 10.1016/j.gene.2008.10.019

[B13] Lee S Morioka T Fujita T (1998) A study on the planning of biotope network with dragonfly as indicator in citizen zone. Environmental Systems Research 26: 617–622. [In Japanese with English abstract]

[B14] Liu B (2011) Web Data Mining: Exploring Hyperlinks, Contents, and Usage Data, Second Edition Springer-Verlag, Berlin, 626 pp.

[B15] Maruyama T (2002) Bass fishing and administrative actions to it. In: Committee for Nature Conservation of the Ichthyological Society of Japan (Ed.) Black Bass: Its Biology and Ecosystem Effects. Koseisha-koseikaku, Tokyo, 99–125. [In Japanese]

[B16] Matsuzawa Y Senou H (2008) Alien Fishes of Japan. Bun-ichi, Tokyo, 157 pp [In Japanese]

[B17] Miyazaki Y (2010) Illegal introduction of largemouth bass (*Micropterus salmoides*) following enactment of the “Invasive Alien Species Act.” Japanese Journal of Ichthyology 57: 86–87. [In Japanese]

[B18] Miyazaki Y Murase A Senou H (2015a) A natural history museum as a platform for accumulating verifiable information on non-native fishes: A Japanese example. Management of Biological Invasions 6: 105–111. doi: 10.3391/mbi.2015.6.1.08

[B19] Miyazaki Y Murase A Shiina M Masui R Senou H (2015b) Integrating and utilizing citizen biodiversity data on the Web for science: An example of a rare triggerfish hybrid image provided by a sport fisherman. Journal of Coastal Research 31: 1035–1039. doi: 10.2112/JCOASTRES-D-14-00170.1

[B20] Miyazaki Y Murase A Shiina M Naoe K Nakashiro R Honda J Yamaide J Senou H (2014) Biological monitoring by citizens using Web-based photographic databases of fishes. Biodiversity and Conservation 23: 2383–2391. doi: 10.1007/s10531-014-0724-4

[B21] Mizuguchi K (2005) Witch-hunt: Why the Black Bass Gets Killed? Furai-no-zasshi, Chofu, 208 pp [In Japanese]

[B22] Nakai K (2002) Bluegill—a strong omnivorous feeder. In: Murakami K Washitani I, The Ecological Society of Japan (Eds) Handbook of Alien Species in Japan. Chijin Shokan, Tokyo, 119. [In Japanese]

[B23] Okiawa H (2010) The Logic of Biodiversity: Quiet Revolution in Japanese Environmental Law. Keiso Shobo, Tokyo, 202 pp [In Japanese]

[B24] Puth LM Post DM (2005) Studying invasions: have we missed the boat? Ecology Letters 8: 715–721. doi: 10.1111/j.1461-0248.2005.00774.x

[B25] Ross S Brennenman WM Slack WT O’Connell MT Peterson TL (2001) The Inland Fishes of Mississippi. University Press of Mississippi, Jackson, Mississippi, xx + 624 pp.

[B26] Saitoh K Uchiyama R (2015) Pictorial Field Guide to Japanese Freshwater Fishes. Yama-kei Publishers, Tokyo, 128 pp [In Japanese]

[B27] Sakai H Yamazaki Y Nazarkin MV Sideleva VG Chmilevsky DA Iguchi K Goto A (2011) Morphological and mtDNA sequence studies searching for the roots of silver crucian carp *Carassius gibelio* (Cyprinidae) from ponds of Sergievka park, Saint Petersburg, Russia. Proceedings of the Zoological Institute Russia Academy of Science 315: 352–364.

[B28] Scott WB Crossman EJ (1973) Freshwater fishes of Canada. Fisheries Research Board of Canada, Bulletin 184, xi + 966 pp.

[B29] Senou H Hayashi M (2013) Centrarchidae. In: Nakabo T (Ed.) Fishes of Japan with Pictorial Keys to the Species, Third Edition Tokai University Press, Hadano, Japan, 820–821 + 1977–1978 [In Japanese]

[B30] Silvertown J Harvey M Greenwood R Dodd M Rosewell J Rebelo T Ansine J McConway K (2015) Crowdsourcing the identification of organisms: A case-study of iSpot. ZooKeys 480: 125–146. doi: 10.3897/zookeys.480.8803 25685027 10.3897/zookeys.480.8803 PMC4319112

[B31] Study Group on Impacts and Managements of Alien Species (2008) Guideline and Case Studies for Alien Species Management in Japanese Riparian Habitats. Foundation for Riverfront Improvement and Restoration, Tokyo, 314 pp [In Japanese]

[B32] Suzuki T Nagano H Kobayashi T Ueno K (2005) Seasonal changes in the number of larvae and juveniles of crucian carps in the reed zone of Lake Biwa based on (sub) species identification using RAPD markers. Nippon Suisan Gakkaishi 71: 10–15. [In Japanese with English abstract]

[B33] Taguchi K Nakagawa M Gouda S Yoshida K Katsurano R Simomoto K (2000) Evalution of swimming pools as biotop using benthos data. Annual Report of Environmental Pollution Control Center, Osaka Prefecture (21): 53–59. [In Japanese with English abstract]

[B34] Takada M Tachihara K Kon T Yamamoto G Iguchi K Miya M Nishida M (2010) Biogeography and evolution of the *Carassius auratus*-complex in East Asia. BMC Evolutionary Biology 10: . doi: 10.1186/1471-2148-10-7 10.1186/1471-2148-10-7 PMC2820001 20064277

[B35] Takeyama H Kamihoshi A Sato H (2002) The plan and design of a biotope at a school based on the behavior of butterflies and dragonflies. Journal of the Japanese Institute of Landscape Architecture 65: 507–512. doi: 10.5632/jila.65.507 [In Japanese with English abstract]

[B36] Taniguchi N (1982) *Carassius* spp. in the western part of Japan featuring the Ō-kin-buna. Tansuigyo 8: 59–68. [In Japanese]

[B37] Tsunoda H Mitsuo Y Senga Y (2011) Illegal stocking of introduced largemouth bass: case studies of irrigation ponds in Oshu City, Iwate Prefecture. Japanese Journal of Conservation Ecology 16: 243–248. [In Japanese with English abstract]

[B38] Yamamoto G Takada M Iguchi K Nishida M (2010) Genetic constitution and phylogenetic relationships of Japanese crucian carps (*Carassius*). Ichthyological Research 57: 215–222. doi: 10.1007/s10228-010-0152-8

[B39] Yamamoto T Yodo T (2014) Bluegill. In: Okiyama M (Ed.) An Atlas of Early Stage Fishes in Japan. Tokai University Press, Hadano, 722–725. [In Japanese]

[B40] Yodo T Iguchi K (2004) A review on the black bass problem referring to the historical background in Japan. Bulletin of Fisheries Research Agency (12): 10–24. [In Japanese with English abstract]

